# Do Older Adults with Multimorbidity Meet the Recommended Levels of Physical Activity? An Analysis of Scottish Health Survey

**DOI:** 10.3390/ijerph16193748

**Published:** 2019-10-04

**Authors:** Ahmad Salman, Maha Sellami

**Affiliations:** 1 Department of Public Health, College of Health Sciences, QU Health, Qatar University, Doha 2713, Qatar; 2 Department of Health Sciences, University of York, Heslington, York YO10 5DD, UK; 3 Sport Science Program (SSP), College of Arts and Sciences (CAS), Qatar University, Doha 2713, Qatar

**Keywords:** physical activity, multimorbidity, older adults

## Abstract

There is a positive association between physical activity (PA) and improved health in older adults. The objective of this study was to assess the prevalence and determinants of meeting recommended levels of PA among older adults with multimorbidity. Data has been derived from the nationally representative Scottish Health Surveys (2014–2017). A sub-sample of 2230 older adults (aged 65+) with multimorbidity were the study participants. Physical activity was evaluated using current recommended guidelines. Overall, 32.3% of the participants met the recommended levels of PA. Independent predictors of meeting the recommended levels of PA include male gender [odds ratio (OR) 2.00 (95% confidence interval (CI) 1.58–2.54)], living in the least deprived areas [OR 1.79 (95% CI 1.20–2.69)]; being a non-smoker [OR 2.22 (95% CI 1.48–3. 34)]. Also, meeting recommended PA decreased with age [OR 0.92 (95% CI 0.90–0.94)] and body mass index [OR 0.93 (95% CI 0.91–0.95]; but increased per additional portion of fruit and vegetables taken [OR 1.19 (95% CI 1.12–1.25)] and with increase in well-being scale score [OR 1.05 (95% CI 1.03 to 1.06)]. Adherence to PA guidelines seems to be more related to age, BMI, gender (i.e. higher PA adherence in men vs. women), social support (i.e. social deprivation), dietary habits (i.e. fruit and vegetable intake) and social isolation among the elderly. In the one-third of older population, adherence to PA was associated to better mental health. Therefore, adaptation of PA guideline to suit theses determinants factors would reduce the gap difference among older adults with multimorbidity and enhance their mental well-being.

## 1. Introduction

Globally, the average life expectancy is increasing over the last three decades [1]. This has led to a rise in the number of people living with chronic diseases or health conditions and the number of years lived with disability [1,2]. Multimorbidity would seem a relatively straightforward term, denoting the co-existence of two or more chronic health conditions in an individual and it is now an important global challenge [3]. A meta-analysis involving over 7 million primary care patients in 12 countries found that the prevalence of multimorbidity ranged from 12.9 to 95.1% [4]. Also, a survey in the United Kingdom has shown that multimorbidity accounts for one-third of all consultations in general practice [5]. The World Health Survey carried out between 2002 and 2004 showed that about 50% of middle-aged adults (i.e. 50–64 years) to elderly (i.e. ≥ 65 years) category have multimorbidity with two or more chronic medical conditions, about a quarter have at least three, and one tenth have four or more chronic medical conditions [6]. Multimorbidity is associated with socioeconomic decline, higher health care utilization and increased risk of poor mental well-being [3,4,5,7]. It has been shown to predict future functional decline, with higher decline in individuals with a higher number of chronic conditions and increased disease severity [7].

Healthy behavior such as physical activity (PA) has been shown to be an important determinant in the management of chronic medical conditions and for the improvement of life expectancy [8,9]. Adherence to recommended PA has been identified as a means of primary and secondary prevention of several chronic diseases such as cardiovascular diseases, diabetes mellitus and cancer [10]. Also, PA has been shown to improve quality of life [11], and it is associated with a 22% reduction in the risk of premature mortality among older adults [12]. Adherence to World Health Organization (WHO) minimum PA levels (i.e. 150 min/week), is associated with a 10% reduction in all-cause mortality, and the WHO estimates that about 3.2 million deaths annually are attributable to physical inactivity [13,14]. Recent studies in several countries have demonstrated that adherence to recommended levels of PA is associated with a significant reduction in chronic diseases risk among adult males and females, and even the practice of once weekly vigorous PA is sufficient to lower the risks of chronic diseases [14,15,16].

Given the benefits on the risk of chronic conditions, the promotion of PA is now considered an essential strategy within the multifaceted care of multimorbidity in the elderly. Studies examining the association between the occurrence of multimorbidity and PA has shown dissenting results with some studies demonstrating an inverse association [17,18] and others reporting no relationship [19,20]. However, one recent multinational study demonstrated that there is a 31% increased risk of multimorbidity among individuals with low PA [6]. Also, a longitudinal study evaluating the burden of multimorbidity over time by levels of PA in elderly English population demonstrated an inverse dose–response relationship with the odds of multimorbidity being lower in those who were physically active than those who were not [21]. Limitations in PA and function due to multimorbidity can substantially affect the treatment burden and response to care among affected individuals, which may further increase multimorbidity [22]. Given the bidirectional interplay between PA and multimorbidity, at the population level, there is limited information on adherence to recommended levels of PA in individuals with multimorbidity and the associated factors. Such information could guide the design and delivery of targeted interventions. We aimed to investigate the prevalence and determinants of meeting recommended levels of PA among older adults with multimorbidity in a Scottish population. 

## 2. Materials and Methods

### 2.1. Study Design and Participants

The analysis is based on a data collected as part of the Scottish Health Survey (SHeS) which is a periodic cross-sectional nationally representative survey commissioned by the Scottish Government Health Directorates [23]. The SHeS was designed to provide comprehensive information about the health and factors related to health of the Scottish population. Further details of the survey’s methods and questionnaire can be found the SHeS website: www.gov.scot/scottishhealthsurvey.

For the purpose of this study, we included data on adults aged 65 and older from SHeS surveys carried out annually between 2014 and 2017 by ScotCen Social Research–Edinburgh, MRC/CSO Social and Public Health Sciences Unit–Glasgow, the Centre for Population Health Sciences- University of Edinburgh and the Public Health Nutrition Research Group–Aberdeen University. This observational study was reported following the guidelines of the Strengthening the Reporting of Observational Studies in Epidemiology (STROBE).

### 2.2. Measures

All participants completed an interviewer-administered questionnaire by trained interviewer. The interview covers anthropometric, longstanding illness, deprivation, smoking, mental health, fruit and vegetable consumption and PA.

Participants were asked about a number of specific health conditions, including doctor- diagnosed diabetes or doctor-diagnosed hypertension and if they had any physical or mental health condition or illness lasting - or likely to last - for twelve months or more [24]. Those who reported having such a condition were asked to provide details of the type(s) of conditions or illnesses reported. For the purpose of this study, we restricted our analysis only to individuals with multimorbidity. Individuals were classified as ‘with multimorbidity’ if they reported two or more longstanding illness. Fruit and vegetable consumption were assessed with the intention to monitor population-level adherence to the five-a-day recommendation. Smoking status was classified into ‘current cigarette smoker’, ‘ex- cigarette smoker’ and ‘never smoked cigarettes at all’. The area deprivation data are presented in Scottish Index of Multiple Deprivation (SIMD) quintiles. Mental well-being was measured using the Warwick-Edinburgh Mental Well-being Scale (WEMWBS) questionnaire [25].

### 2.3. Physical Activity

Physical activity interview module questions were based on the Allied Dunbar National Fitness Survey [24]. Participants were asked to report any activities that lasted at least 10 minutes and the number of days in the past four weeks in which they had taken part in such activities. The interview questions consisted of four main types of physical activity: home-based activities (housework, gardening, building work and DIY), walking, sports and exercise, and activity at work. The information was collected based on time spent being active, intensity of the activities undertaken, and frequencies with which activities are performed. The intensity level of activities mentioned by participants was estimated to help assess adherence to this guideline. A more detailed discussion of estimation of the amount of PA is provided in the SHeS report [24]. Physical activity was classified into ‘Met’ and ‘Unmet’ based on Moderate or Vigorous PA (MVPA) guidelines on the recommended amounts of PA for adults, issued by the UK Chief Medical Officer in 2011 [26]. Current guidelines in the UK recommends that adults need to undertake 150 minutes of moderate PA per week or 75 minutes of vigorous activity per week or an equivalent combination of both [24]. This is the recommended level of PA referred to throughout this paper.

### 2.4. Ethical Considerations

The SHeS data were analysed with the permission of the UK Data Service [23]. The cross-sectional surveys data are freely available for academic researchers to download from the UK Data Service. Participants gave informed consent for data linkage for future research data analysis

### 2.5. Statistical Data Analysis

The data analysis in this study was performed with IBM Statistical Package and Services Solutions (SPSS) software version 25 (IBM Corp, Armonk, New York, USA). A p value ≤0.05 was considered statistically significant. Categorical variables were summarized as proportion and continuous variables were reported as mean (SD: standard deviation). The main outcome variable was meeting the recommended PA. A univariate analysis was first performed to determine the association between the outcome variable and potential predictor variables. The t test (or where appropriate ANOVA) was used to compare continuous variables. Logistic regression analysis was performed to assess the association between meeting recommended PA in older adults with sociodemographic and clinical factors and including all variables with univariate p = 0.1 or lower to determine the independent predictors of the outcome variable. Model fit was assessed using the likelihood ratio test. The area under the ROC curve was 0.74, 95% CI [0.71, 0.77], which is an acceptable level of discrimination according to Hosmer et al. [26]. The logistic regression model was statistically significant (χ^2^ (11) = 258.80, *p* < 0.0001), explained 21.8% (Nagelkerke R2) of variance in meeting recommended PA, and correctly classified 69.9% of cases. Sensitivity was 49.6%, specificity 82.7%, positive predictive value 64.3%, and negative predictive value 72.3%. 

## 3. Results

Of the total 5106 adults aged 65 years and over [2287 men (44.8%) and 2819 women (55.2%)], who participated in the 2014–2017 period, total numbers of valid samples that have been used in this analysis were 2230 who have two or more morbidities. The morbidity profile of the participants is summarized in Table 1. The commonest morbidities among the participants were arthritis (36.6%), hypertension (30.0%) and diabetes (18.8%). To assess the model for influential cases, Cook’s distance test and leverage values were computed but neither test produced unusually high values (*p* < 1.00 for all). The Hosmer–Lemeshow test in the final model was not statistically significant (*p* = 0.56), indicating that the model is not a poor fit.

The characteristics of the participants are summarized in Table 2. Mean age of the participants was 74.54 (6.87) years and their mean body mass index (BMI) was 29.29 (5.53) kg/m^2^. Overall, 32.3% of the participants met the recommended levels of PA, and 18.3% were in the most-deprived quintile. Also, 12.4% of them were current smokers. The average total portion of fruit and vegetable intake per participant was 3.12 (2.18) portions. 

The differences in the participants meeting the recommended PA according to their demographic characteristics are summarized in Table 3 and Table 4. The proportion of current smoker among those meeting and not meeting the recommended PA levels was (9.1% vs. 14.1%; *P* < 0.05). Also, there was a progressive increase in the proportion of deprivation among participants not meeting the recommended PA level (*p* < 0.05). The mean total portion of fruit and vegetable intake per week among participants meeting and not meeting the recommended PA level were (3.81 ± 2.30 vs. 2.80 ± 2.05; *p* < 0.05). The mean age (72.28 ± 5.87 vs. 75.64 ± 7.06 years; *p* < 0.05) and BMI (28.63 ± 4.61 vs. 29.68 ± 5.98 kg/m^2^; *p* < 0.05) of the participants meeting the recommended PA level was significantly lower than those not meeting the recommended PA level. However, there was a significantly higher mean WEMWBS score among participants meeting the recommended level of PA (51.66 ± 7.95 vs. 47.67 ± 8.42; *p* < 0.05).

A binomial logistic regression was performed to ascertain sociodemographic and clinical factors associated with meeting recommended PA in older adults with multiple comorbidities (Table 5). The probability of meeting recommended PA was 100% higher (odds ratio (OR) 2.00 (95% CI 1.58 to 2.54)) for male than female; 79% higher (OR 1.79 (95% CI 1.20 to 2.69) for those living in the least deprived areas than among those in the most deprived areas; 122% higher (OR 2.22 (95% CI 1.48 to 3.34) for never smoked at all or never regular smoker than current smoker.

The probability of meeting recommended PA decreased by 8% (OR 0.92 (95% CI 0.90 to 0.94)) per additional year and by 7% (OR 0.93, 95% CI 0.91 to 0.95) per 1-point increase in BMI.

The probability of meeting recommended PA increased by 19% (OR 1.19 (95% CI 1.12 to 1.25)) per additional portion of fruit and vegetables and by 5% (OR 1.05, 95% CI 1.03 to 1.06) per 1-point increase in WEMWBS score.

## 4. Discussion

The current study aimed to assess the prevalence and determinants of meeting recommended PA level among Scottish older adults with multimorbidity. The findings of the study showed that about one-third of the study population were adherent to the recommended PA levels, and a slightly higher proportion of men than women met the recommended level of PA. The independent predictors of meeting recommended PA levels were male gender, living in less-deprived areas, being a non- or ex-smoker, having lower BMI, higher fruit and vegetables intake and higher WEMWBS score. Together, the findings of this study suggest that there are opportunities for a health promotion intervention to modify the lifestyle of Scottish older adults towards improving the outcomes of their co-morbid chronic medical conditions. 

Despite the substantial evidence on the inverse relationships between PA and the risk of development or worsening of the cardiovascular diseases [27,28,29], we found that less than a third of the study population with multimorbidity met the recommended PA level. This high nonadherence to recommended PA will most likely predispose them to either worsening of the multimorbidity or have poor management outcomes. In this study, we found that belonging to the less-deprived socioeconomic groups was an independent predictor of meeting the recommended level of PA among older adults with multimorbidity surveyed. Our finding is consistent with the results of several studies, reviews and their umbrella review which showed that older adults with higher socioeconomic status are more likely to undertake PA compared with those belonging to the lower socioeconomic group [30,31,32,33]. This suggests that improving the socioeconomic status of the elderly in this setting may improve their likelihood of adopting healthy behaviours like PA. A cohort study in Germany showed that older adults with high social support are more likely to undertake PA compared with those with low social support [31].

In this study, we found that non-modifiable factors like age and gender were also predictors of PA in the elderly with multimorbidity. We found that there was an inverse association between age and PA. This suggests that even among the elderly, the older they become the less likely they may meet the recommended level of PA. Possible reasons may be that most elderly individuals are prone to substantial disability, gait disorder and falls during late life leading to activity restriction [33,34]. Also, we found that male participants were twice more likely to meet the recommended PA levels compared with females. Previous studies have documented gender differences in the levels of PA among the elderly with men significantly more likely to engage in sporting activities and women significantly more likely to engage in domestic activities [35,36,37].

Being a non- or an ex-smoker was an independent predictor of meeting the recommended level of PA in this study. This suggests that not smoking or smoking cessation potentially has other health benefits. Previous studies among older adults on the relation between smoking and PA have provided mixed findings; while majority of the studies found that there was a significant relationship between being a non- or an ex-smoker and higher PA [38,39], a few found no relationship between smoking and PA [31]. The differences between these studies deserve further investigation. Furthermore, a previous study has shown that overweight and obese individuals with low PA had a higher cardiovascular disease risk than normal weight participants with high PA [40]. This underscores the importance of PA in health outcomes of older adults with multimorbidity. In this study, we found an inverse association between BMI and physical activity. This is consistent with previous studies and suggest the need for weight reduction among individuals with multimorbidity [41,42]. Also, we found that higher fruits and vegetable consumption was associated with meeting recommended PA. This is consistent with previous studies that showed that higher fruits and vegetable consumption was associated with good self-rated health and the likelihood to engage in healthy behaviours [43,44]. 

This study also showed that having a higher degree of mental well-being was a predictor of meeting recommended PA among older adults with multimorbidity. This finding adds to the growing body of evidence on the relationship between mental well-being and PA. Several studies have shown that there is a direct relationship between PA and mental well-being in the elderly [45,46,47]. A study in the United States found that light physical activities were positively associated with mental health and life satisfaction in summer [45]. However, engaging in moderate physical activities was positively related only with physical health [44]. Other population-based studies showed a consistent association between enhanced psychological well-being, as measured using a variety of psychological inventories, and regular physical exercise [46,47]. Our study indicates that there is likelihood of a bidirectional relationship between PA and mental well-being. Physical activity and especially moderate intensive aerobic exercise is shown to be accompanied by higher benefits in well-being and mood [48], and quality of life [49]. In the elderly, these activities that require oxygen (brisk walking, running, swimming) also allow them to build their brains [50]. In this study researchers explain that two sessions a week could prevent age-related neurological decline and even strengthen the memory of older people. The aerobic exercise for 70 to 80% of the maximum cardiac capacity promote oxygen flow to the muscles, it helps to work the heart and lungs.

This study has the following strengths. First, it was based on data from the elderly with multimorbidity complemented by self-reported assessment of PA and mental well-being. Second, it was based on data from large, nationally representative surveys designed to measure health status at the population level. However, the study has some limitations. All analyses performed were cross-sectional and causal inferences should not be made. Second, some of the information collected during the survey may be limited by recall bias. Third, some of the assessment made e.g. the relationship between PA and mental well-being is likely to be moderated by seasonal variations [43]. We did not adjust for the effect of season in our regression model. Further research should consider adjusting for additional indicators such as social support, season, dietary habits and social isolation among the elderly.

## 5. Conclusions

About one-third of older Scottish population with multimorbidity had recommended PA levels, with a slight male preponderance. Being male, living in less-deprived areas, being a non- or ex-smoker, having lower BMI, have higher fruit and vegetables intake and higher mental well-being score were determinants of meeting recommended PA in the study setting. Multi-level and multi-component interventions targeting the identified determinants are needed to increase PA in the older adults with multimorbidity in the study setting.

## Figures and Tables

**Table 1:** Morbidities profile of older adults with multimorbidity.

Characteristics	% (N)	Male %	Female %
Cancer	9.1% (204)	48%	52%
Diabetes	18.8% (420)	49.3%	50.7%
Mental disorders	8% (179)	37.4%	62.6%
Stroke	3.8% (85)	37.6%	62.4%
Ischaemic heart disease/Heart attack/angina	12.6% (280)	45.7%	54.3%
Hypertension	30% (670)	43%	57%
COPD	9% (201)	49.8%	50.2%
Asthma	9.2% (205)	35.1%	64.9%
Arthritis/rheumatism/fibrositis	36.6% (817)	33.8%	66.2%
Back problems/slipped disc/spine/neck	11.6% (259)	44.8%	55.2%

COPD, Chronic Obstructive Pulmonary Disease; N, number of older adults; %, percentage of morbidity.

**Table 2:** Demographics and health states of older adults with multimorbidity.

Characteristics		Male	Female
PA meeting recommendations	Met (32.3 %)	52.3%	47.7%
	Unmet (67.7%)	37.9%	62.1%
SIMD quintiles	Least deprived (17.4%)	43.3%	56.7%
	4 (19.2%)	42.8%	57.2%
	3 (23.5%)	43.9%	56.1%
	2 (21.6%)	42.7%	57.3%
	Most deprived (18.3%)	40%	60%
Cigarette smoking status	Never smoked (44.5%)	32.2%	67.8%
	Ex-smoker (43.1%)	52.2%	47.8%
	Current smoker (12.4%)	46.6%	53.4%
Mean (SD) Age	74.54 (6.87)	74.13 (6.62)	74.85 (7.04)
Mean (SD) BMI	29.29 (5.53)	29.58 (5.25)	29.07 (5.73)
Mean (SD) portion of fruit and vegetable	3.12 (2.18)	3.11 (2.21)	3.13 (2.17)
Mean (SD) WEMWBS score	49.06 (8.48)	48.79 (8.58)	49.26 (8.41)

BMI, body mass index; PA, Physical activity; SD, standard deviation; SIMD, Scottish Index of Multiple Deprivation; WEMWBS, Warwick-Edinburgh Mental Well-being Scale.

**Table 3:** Difference of categories variables between groups.

Characteristics		Meeting PA Group %	Unmet PA Group %
Cigarette Smoking Status *	Never smoker	48.3%	42.7%
	Ex-smoker	42.7%	43.2%
	Current smoker	9.1%	14.1%
SIMD *	Least deprived	22.3%	15%
	4	23.4%	17.3%
	3	22.7%	23.8%
	2	18.3%	23.2%
	Most deprived	13.2%	20.7%

PA, physical activity; SIMD, Scottish Index of Multiple Deprivation; * *p* ≤ 0.05.

**Table 4:** Difference of continuous variables among groups.

Characteristics	Meeting PA Group; Mean (SD)	Unmet PA Group; Mean (SD)
Age *	72.28 (5.87)	75.64 (7.06)
BMI *	28.63 (4.61)	29.68 (5.98)
Total portion of fruit and vegetables *	3.81 (2.30)	2.80 (2.05)
WEMWBS score *	51.66 (7.95)	47.67 (8.42)

BMI, body mass index; PA, Physical activity; SD, standard deviation; WEMWBS, Warwick-Edinburgh Mental Well-being Scale; * *p* ≤ 0.05.

**Table 5:** Full details on binomial logistic regression.

	B	SE	Wald	df	p	OR	95% CI for OR	
							Lower	Upper
Gender (Male)	0.69	0.12	32.92	1.00	0.000 *	2.00	1.58	2.54
SIMD (least deprived)	0.58	0.21	7.96	1.00	0.005 *	1.79	1.20	2.69
SIMD (4)	0.66	0.20	10.88	1.00	0.001 *	1.94	1.31	2.88
SIMD (3)	0.29	0.20	2.17	1.00	0.140	1.33	0.91	1.96
SIMD (2)	0.54	0.20	7.14	1.00	0.008 *	1.72	1.15	2.55
SIMD (most deprived as reference)			14.35	4.00	0.006 *			
Age	−0.09	0.01	72.12	1.00	0.000 *	0.92	0.90	0.94
Smoking status (never smoker)	0.80	0.21	14.65	1.00	0.000 *	2.22	1.48	3.34
Smoking status (ex-regular smoker)	0.54	0.21	6.95	1.00	0.008 *	1.72	1.15	2.59
Smoking status (current smoker as reference)			15.30	2.00	0.000 *			
Total portion of fruit and vegetables	0.17	0.03	35.81	1.00	0.000 *	1.19	1.12	1.25
BMI	-0.07	0.01	34.80	1.00	0.000 *	0.93	0.91	0.95
WEMWBS score	0.05	0.01	38.09	1.00	0.000 *	1.05	1.03	1.06
Constant	3.79	0.94	16.36	1.00	0.000 *	44.10		

B = unstandardised regression coefficient; BMI, body mass index; CI = Confidence Interval for odds ratio; df, degrees of freedom; OR, odds ratio. S.E.; standard error of the coefficient; SIMD, Scottish Index of Multiple Deprivation; WEMWBS, Warwick-Edinburgh Mental Well-being Scale; * *p* ≤ 0.05.
